# An Example of Using Low-Cost LiDAR Technology for 3D Modeling and Assessment of Degradation of Heritage Structures and Buildings

**DOI:** 10.3390/ma17225445

**Published:** 2024-11-07

**Authors:** Piotr Kędziorski, Marcin Jagoda, Paweł Tysiąc, Jacek Katzer

**Affiliations:** 1 Faculty of Civil Engineering, Environmental and Geodetic Sciences, Koszalin University of Technology, Śniadeckich 2, 75-453 Koszalin, Polandmarcin.jagoda@tu.koszalin.pl (M.J.); 2 Faculty of Civil and Environmental Engineering, Gdańsk University of Technology, Gabriela Narutowicza 11/12, 80-233 Gdańsk, Poland; pawel.tysiac@pg.edu.pl; 3 Faculty of Geoengineering, University of Warmia and Mazury in Olsztyn, Prawocheńskiego 15, 10-720 Olsztyn, Poland

**Keywords:** low-cost LiDAR, 3D modeling, terrestrial laser scanning (TLS), heritage conservation, mobile LiDAR, historic building documentation

## Abstract

This article examines the potential of low-cost LiDAR technology for 3D modeling and assessment of the degradation of historic buildings, using a section of the Koszalin city walls in Poland as a case study. Traditional terrestrial laser scanning (TLS) offers high accuracy but is expensive. The study assessed whether more accessible LiDAR options, such as those integrated with mobile devices such as the Apple iPad Pro, can serve as viable alternatives. This study was conducted in two phases—first assessing measurement accuracy and then assessing degradation detection—using tools such as the FreeScan Combo scanner and the Z+F 5016 IMAGER TLS. The results show that, while low-cost LiDAR is suitable for small-scale documentation, its accuracy decreases for larger, complex structures compared to TLS. Despite these limitations, this study suggests that low-cost LiDAR can reduce costs and improve access to heritage conservation, although further development of mobile applications is recommended.

## 1. Introduction

The progressive development of survey technologies, especially LiDAR technology, has brought significant changes in the way historic sites are inventoried and documented [1,2,3]. Traditionally used methods, such as stationary terrestrial laser scanning (TLS), have high accuracy, but their use is associated with high equipment costs and a time-consuming survey process. In response to these challenges, there has been a growing interest in recent years in low-cost LiDAR technologies that offer more accessible and rapid solutions [4,5,6,7].

One of the most important innovations in this field is the integration of LiDAR scanners with mobile devices, such as Apple’s iPad Pro, which have been unveiled as tools available to a wider range of users. The technology, based on solid-state LiDAR (SSL) and direct time of flight (DToF), offers the ability to acquire 3D point clouds from mobile devices, making it an attractive alternative to more expensive solutions. Although the technical specifications of LiDAR on Apple devices are protected by trade secrets, reverse engineering studies [8] have shown that the technology, while not on par with professional TLS scanners, offers sufficient accuracy for many inventory tasks [9,10,11,12].

The literature dedicated to low-cost LiDAR scanners is focused on exploring their capabilities and limitations in the context of architectural and archeological documentation. Teppati Losè et al. [13] demonstrated that LiDAR scanners integrated with mobile devices can effectively map the geometric properties of objects, especially for smaller objects. Murtiyoso et al. [7] demonstrated the potential of using Apple LiDAR to create documentation about historic objects. They outlined the importance of low-cost survey equipment in the often-underfunded field of cultural heritage. The advantages of low-cost measurement technology were also presented by Waliulu et al. [14], pointing out in their research its great potential in determining the volume of cavities in road surfaces. Studies completed to date show that LiDAR technology provided by Apple offers the potential to reduce survey costs and simplify related work. However, not all researchers agree on the full usefulness of this technology in the context of historic preservation. Blaszczak-Bąk et al. [15] and Purfürst et al. [16] highlight significant challenges related to the accuracy of measurements taken by LiDAR scanners on mobile devices. Their analyses suggest that for larger sites with more complex geometry, low-cost technologies can generate significant errors, limiting their application to selected monument types.

In light of the above studies, a gap was noted in the realized analyses, motivating the need to develop the topic of the usefulness of low-cost survey technologies in inventory studies of historical sites. Thus far, the authors have focused on the one-time use of low-cost LIDAR technology and its comparison with TLS data. In this paper, an approach to detect the deterioration over time through two measurements of an object made within a one-year interval is presented and analyzed. The purpose of this work is to investigate the feasibility of using low-cost LiDAR technologies for detecting the degradation of historic objects. In particular, the potential of the method in the context of documenting smaller architectural fragments and identifying the limitations of using these tools in conservation practice was assessed.

## 2. Research Area

**Figure 1:**
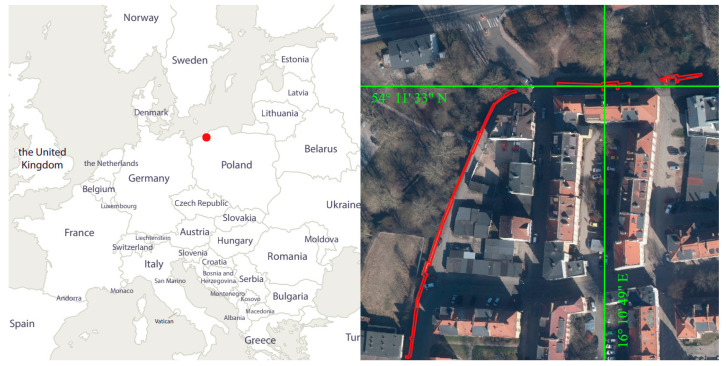
Location of the object under study.

The object under study is a section of the historic city walls of Koszalin located in Poland in the West Pomeranian Voivodeship. Its location is shown in Figure 1. The red dot marks the location of the city of Koszalin on the map of Europe.

**Figure 2:**
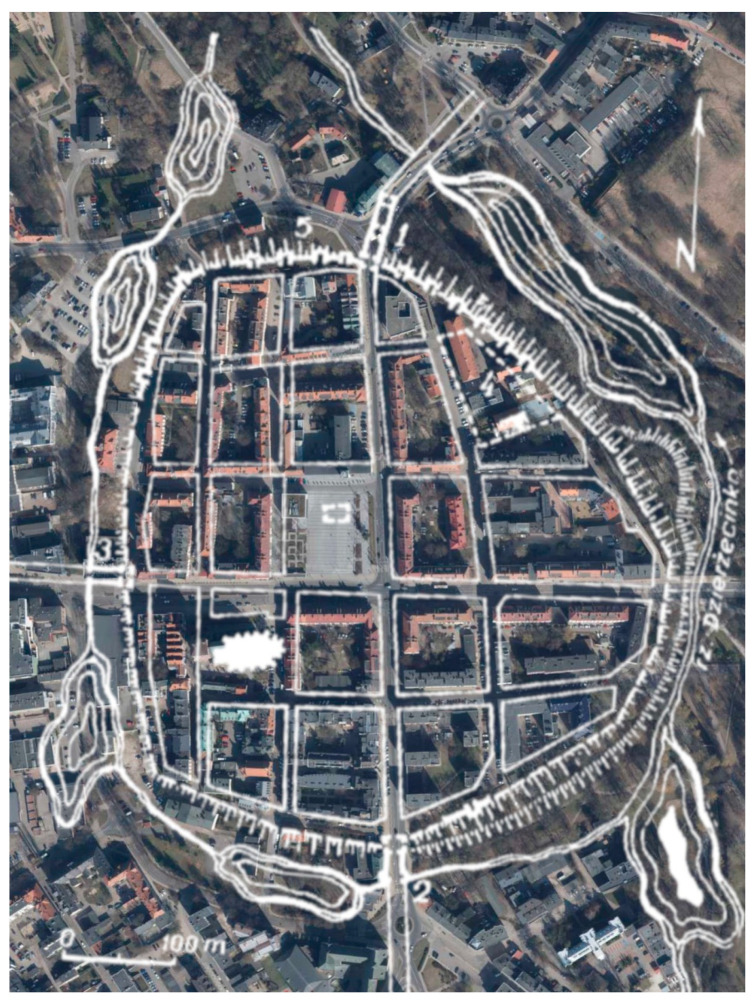
City plan with the existing wall sections plotted on a current orthophotomap [17].

**Figure 3:**
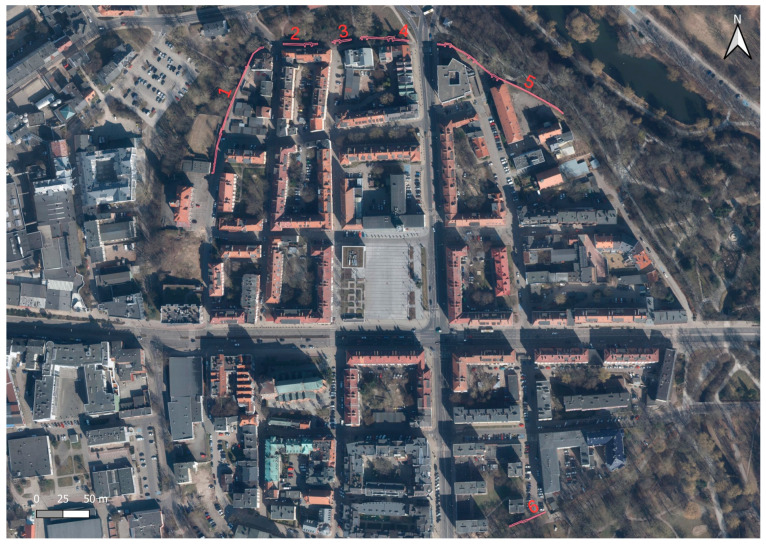
Six fragments of walls that survive today, numbered from 1 to 6.

The first mention of the city of Koszalin in written sources dates back to 1214. The first city walls were built in 1266 and were only a system of earthen ramparts with a wooden palisade surrounded by a moat and ponds. At the end of the 12th century, construction of Koszalin’s city walls began, and work continued into the early 14th century. In 1310, the city council obliged the Koszalin Cistercian monastery to build a wall adjacent to the monastery. The resulting walls were rebuilt and expanded. In the 14th century, the city walls, with a circumference of about 1560 m and a height of more than 7 m, had three gates and 46 towers. It was built of brick and mortar, with stone used as infill and for the foundation. Figure 2 shows a plan of the city with marked existing parts of the walls. Until the 18th century, the city walls were in good condition. In 1718, the city was destroyed by fire, which caused the gradual lowering of the walls (which were no longer of military importance), and the reclaimed bricks were used to rebuild the city. Initially, the walls were lowered to a height of 6 m. In 1731, one of the three gates was adopted as the city jail. In subsequent years, the walls were gradually dismantled. The New Gate was dismantled in 1819, the High Gate in 1867, the Mill Gate in 1872, and the Powder Tower in 1900. The preserved sections of the wall survived due to the fact that residential houses were added to them. After the destruction suffered by the city during World War II, the walls were exposed. Their poor technical condition called for quick restoration work. In 1960–1968, the first works were carried out, and, since then, the walls have been successively renovated [17,18]. The remains of Koszalin’s city walls were entered in the register of monuments on 25 May 1955. The location of five still-existing sections of the walls is shown in Figure 3. For the purpose of this article, Section 2 was selected for this study. This part of the wall contains the remains of the tower. This section, which is about 33 m long, has a low height of about 1 m, while the remains of the tower are about 1.5 m high.

## 3. Methodology

**Figure 4:**
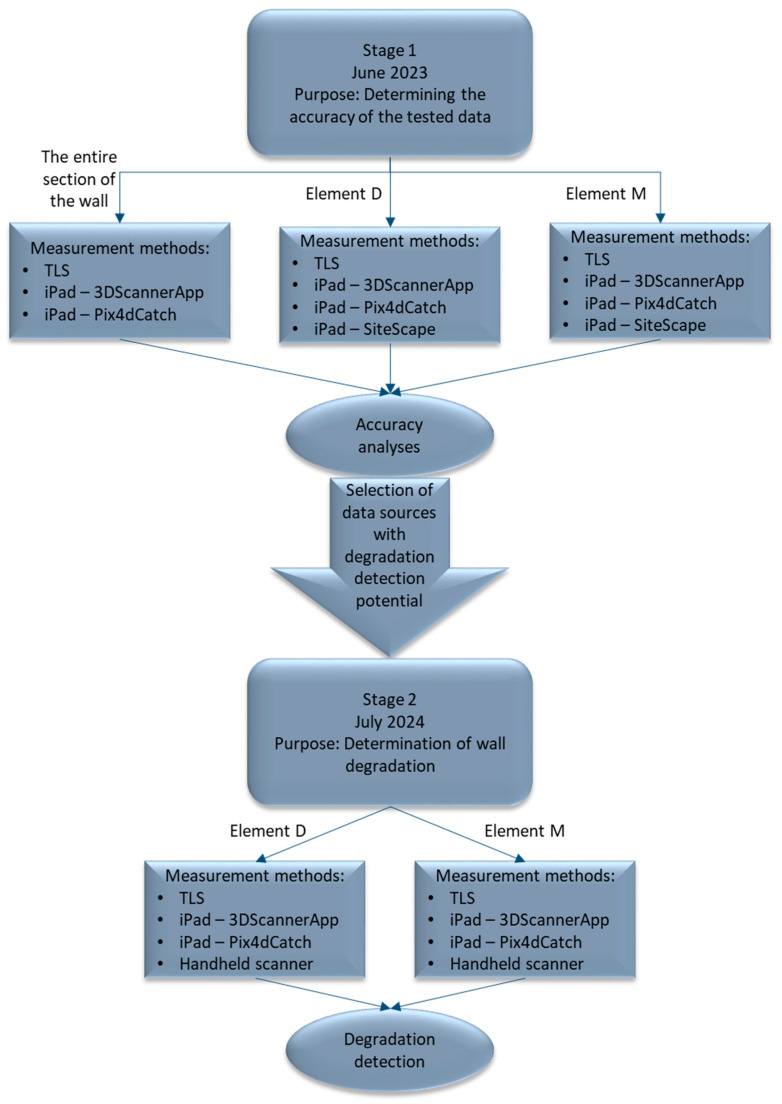
Workflow of the research program.

The research presented in this article was divided into two stages. Stage 1 aimed to determine the accuracy of the selected measurement methods, and the second tested whether the methods would detect object degradation. The measurement tools used were TLS, an iPad Pro (2nd generation) with a low-volume scanner by Apple Inc. (Cupertino, CA, USA), and a FreeScan Combo handheld scanner from SHINING 3D (Hangzhou, China). The measurements were carried out in June 2023. Stage 2 of the research program was conducted after a one-year time interval (June 2024). Its main purpose was to assess the degradation of the city walls. The full workflow of the research program is shown in Figure 4.

### 3.1. Measurement Equipment Used

The 2nd-generation iPad Pro developed by Apple Inc. (Cupertino, CA, USA) has an 11-inch Liquid Retina display, weighs 471 g, has 128 GB of storage, has a 6-core A10X Fusion processor, has 4 GB of RAM, and runs iOS version 16.6.1. The technical specifications of the LiDAR scanner used in Apple’s devices are kept secret by the manufacturer due to intellectual property protection. However, reverse-engineering analyses have shown that they use a solid-state lidar (SSL) scanner, which dispenses with moving mechanical parts [7,8,13,19]. The scanner is based on direct time-of-flight (DToF) technology, which measures distance through direct time of flight. The viewing angle of the DToF system on Apple devices is approximately 60° × 48°. The scanner uses a VCSEL emitter that generates 16 rows of 4 points each. The grid of 64 points thus created is then duplicated by the DOF element in four directions: vertical, horizontal, and two diagonal directions. The end result is a matrix of 9 basic segments, containing 567 strands [20]. This innovative approach makes it possible to obtain a larger number of measurement points with the compact size of the instrument. Signals are received by a SPAD (single-photon avalanche diode), which provides precise measurements at the single-photon level and integration with CMOS arrays [13,21,22]. Three applications supporting LiDAR scanning are installed on the device: 3DScannerApp (v. 2.0.15), SiteScape (v. 1.7.11), and PIX4DCatch (v. 1.28.0). All apps are provided to users free of charge. SiteScape allows users to purchase the Pro version, allowing measurement of larger areas. For the purposes of the research program, the basic version of the app was used. Pix4DCatch is an application for acquiring photogrammetric data, but it also provides the possibility of measurements using LiDAR. The application does not allow the direct export of survey data. They must first be post-processed in a paid desktop application.

The FreeScan Combo handheld scanner from Shining 3D (Hangzhou, China) is a handheld scanner designed for precision metrology applications. The device measures 193 × 63 × 53 mm and weighs 620 g, making it a small, portable device suitable for use even in narrow spaces. The scanner is equipped with dual-light sources: a blue laser and an infrared VCSEL (vertical-cavity surface-emitting laser). This combination allows the FreeScan Combo to work in two different modes. The blue light mode allows for a measurement accuracy of 0.02 mm at a speed of up to 3,600,000 points per second. The distance between points in the point cloud can be adjusted from 0.05 to 10 mm. Additionally, it offers 3 scanning methods. The multi-line method uses 26 laser lines, which allows for the fast scanning of large objects. The precision method uses 7 parallel laser lines, ideal for capturing intricate details. For more demanding geometry, the deep pocket scanning method uses a single laser line, designed specifically to reach deep pockets and complex areas. When performing measurements in this mode, special measurement marks must be placed on the object. They must be placed quite densely because, during the measurement, the scanner must see at least 3 such marks in each position. The second mode uses infrared light. It is characterized by an accuracy of 0.05 mm and a speed of 2,250,000 points per second. The distance between points in the point cloud can be adjusted from 0.1 to 3 mm. This mode has a lower accuracy and scanning speed but does not require the use of measurement marks. Subsequent measurement scenes are combined with each other using real-time registration algorithms.

**Figure 5:**
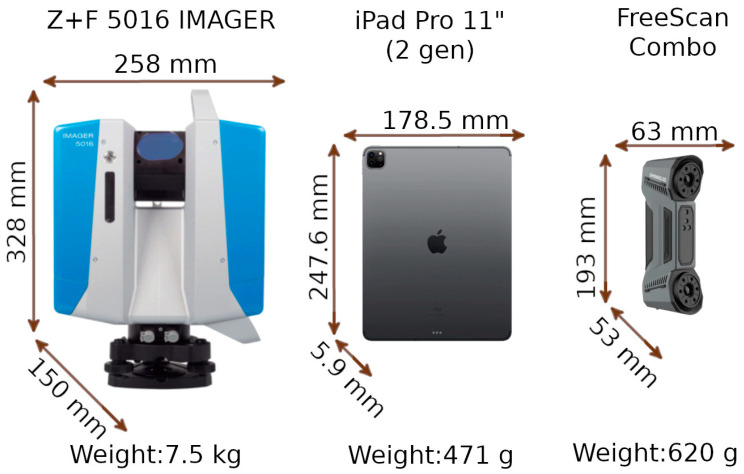
Dimensions and weights of the equipment used.

In this study, the Z+F 5016 IMAGER terrestrial laser scanner from Zoller & Fröhlich (Wangen im Allgäu, Germany) was employed. The device is characterized by phase-shift measurement capabilities (acquiring data at a rate of up to 1,860,000 points per second, with an operational range extending from 0.3 to 365 m). This range enables the device to be utilized in both short- and long-range measurement contexts, including applications in architectural surveying, civil engineering, and environmental monitoring. The scanner’s mean positional error is specified as ≤1 mm + 10 ppm/m, reflecting the device’s capacity to maintain positional accuracy over varying distances. The phase-shift technology incorporated within the Z+F 5016 functions by analyzing the phase difference between emitted and received laser signals, which facilitates the collection of measurement data in environments with heterogeneous reflectivity and complex surface geometries. Additionally, the scanner integrates several functionalities, including high-dynamic-range (HDR) imaging, which augments the detail captured during scans, and an integrated laser plummet, aiding in the precise alignment and setup of the device. Furthermore, the Z+F 5016 IMAGER includes on-device data processing capabilities, which support real-time point cloud registration and visualization. This onboard processing feature reduces the need for extensive post-processing and enhances the efficiency of the data acquisition process. A graphical comparison of the sizes and weights of the devices used is shown in Figure 5.

### 3.2. Measurements

**Figure 6:**
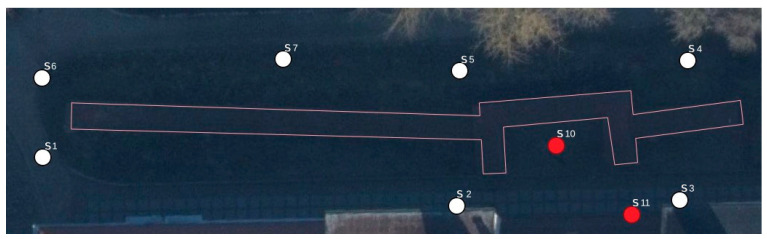
Locations of scanner position.

**Figure 7:**
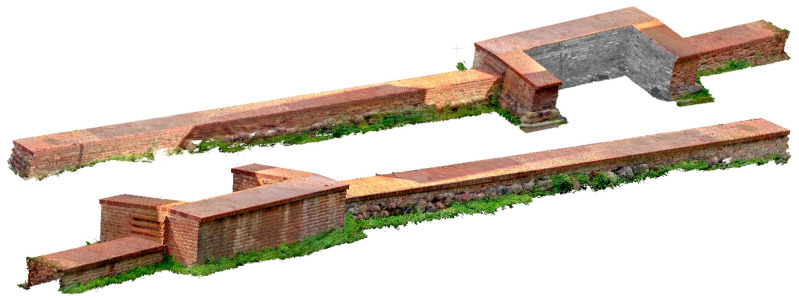
Achieved point clouds using TLS.

In Stage 1, several measurements were carried out. The first involved the measurement of an entire section of the city walls using a TLS Z+F 5016 IMAGER terrestrial scanner. In further work, this measurement served as a reference method for evaluating the accuracy of the other measurement methods. It consisted of nine sites, the location of which, in relation to the wall, is shown in Figure 6. Positions marked in red indicate sites from which the accuracy of smaller fragment of the city walls will be analyzed later in the article. Figure 7 shows the point cloud acquired from the TLS.

The same section of the wall was also measured with low-cost LiDAR scanners, using 3DScannerApp and Pix4DCatch. The measurement was made by maintaining a distance of about 1 m between the device and the measured object. The SiteScape app did not allow measurement of the entire section of the wall due to exceeding the maximum number of points that could be recorded.

**Figure 8:**
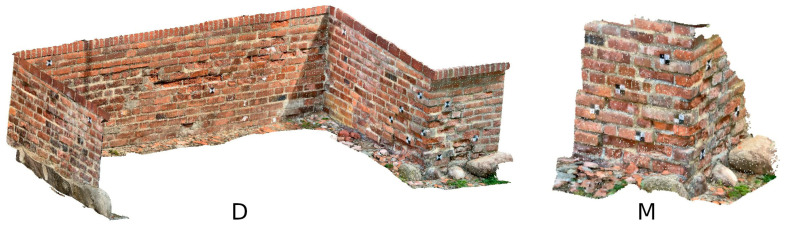
Measurement results from 3DScannerApp for fragment D and M.

In addition, two sets of measured data were collected, showing fragments of the wall characterized by significant erosion. These were measured with an iPad Pro using 3DScannerApp, SiteScape, and Pix4DCatch. The larger fragment of the wall (labeled D) was measured from a distance of about 0.8 m, while the smaller fragment (labeled M) was measured from a distance of about 0.3 m. Example point clouds, labeled D and M, are shown in Figure 8.

In Stage 2, the data set for the entire section of the wall was measured using only the terrestrial scanner, as the other methods did not perform well. Measurement errors of low-cost data sources reached as high as 51 cm. More detailed analyses are presented later in this article.

City wall fragments labeled D and M were again measured with an iPad Pro, using the 3DScannerApp and Pix4DCatch applications. The SiteScape app, due to its low accuracy (as found in Stage 1), was no longer used in Stage 2.

**Figure 9:**
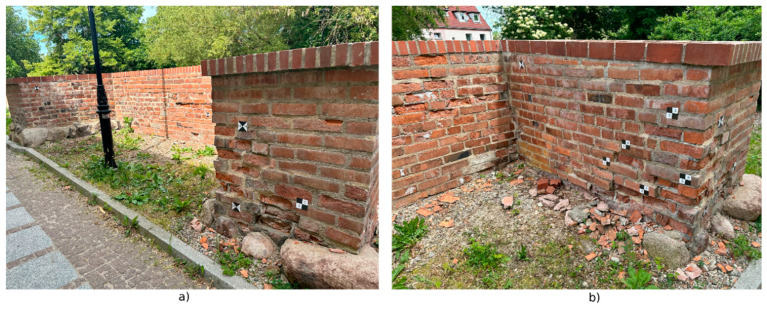
Location of selected measurement markers. (**a**). View of fragment D. (**b**). View of fragment M.

Stage 2 of the survey additionally introduced a new measurement method based on the FreeScan Combo handheld LiDAR scanner. This scanner was used to measure D and M fragments, using an infrared mode that eliminated the need for additional survey markers. The data acquired in this way were processed using the CloudCompare application. To facilitate the registration of the scans to a common coordinate system, survey markers were used, as shown in Figure 9. Once the common coordinate system was assigned, the data were cleaned of unnecessary observations and then further analyzed.

## 4. Results

### 4.1. Stage 1

The data acquired with the TLS Z+F 5016 IMAGER terrestrial scanner served as reference data for evaluating the accuracy and quality of the results obtained from the low-cost LiDAR scanner. They were characterized by high density and accuracy, making them the most reliable data set in this study.

The first set to be compared were point clouds representing the entire section of the city wall selected for the survey. As mentioned, measurements with a low-cost LiDAR scanner were taken using 3DScannerApp and Pix4DCatch. The SiteScape application proved to be inadequate for this section due to its excessive size, which would have required dividing the wall into smaller fragments and post-processing them (which was not the purpose of this part of this study).

The 3DScannerApp data were the easiest to acquire, and after a brief post-processing directly in the app, it was exported in .las format. For the Pix4DCatch application, two options were available. The first allowed the export of the acquired point clouds without further processing—these data were labeled Pix4D Captured. The second option required further processing of the data. For this purpose, the Pix4DMatic application was used, which allows two variants of data processing: Depth—data collected by the LiDAR scanner subjected to advanced post-processing in Pix4DMatic; and Fused—LiDAR data combined with close-range photogrammetry, also subjected to post-processing.

**Figure 10:**
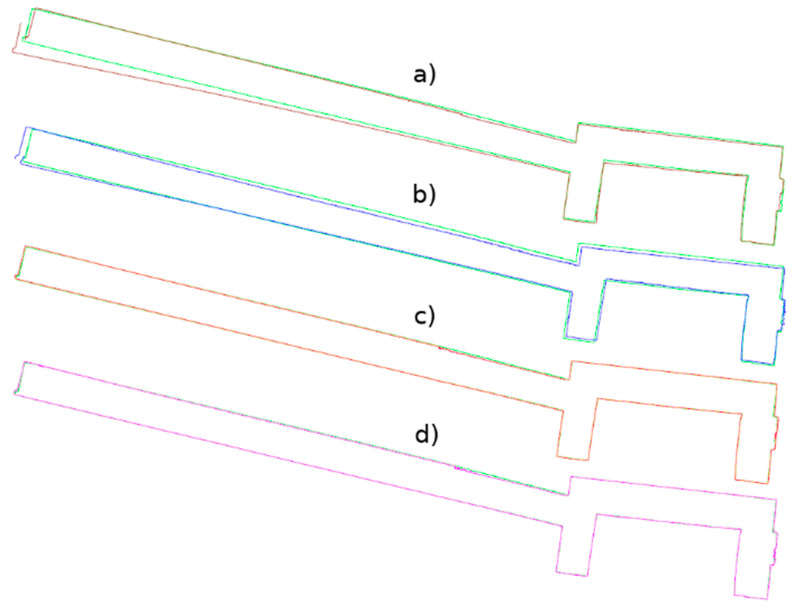
Cross-section through the acquired point clouds in relation to the reference cloud (green): (**a**). 3DScannerApp; (**b**). Pix4DCatch Captured; (**c**). Pix4DCatch Depth; (**d**). Pix4DCatch Fused.

This resulted in five datasets for the entire wall: the TLS reference data, data from the 3DScannerApp, and three datasets from Pix4DCatch: Captured, Depth, and Fused. Figure 10 shows the horizontal cross-sections of each point cloud, juxtaposed with the reference cloud.

As shown in Figure 10, the data not subjected to post-processing in the desktop applications (a, b) show a significant measurement error in the left part of the wall, where the scanning process started and ended. For 3DScannerApp, the maximum error in this part of the wall was 51 cm, while that for Pix4D Captured was 28 cm. In the post-processed data, this error did not occur. Despite some irregularities in the geometry, most of the city walls were imaged with much higher accuracy. In Table A1, the results of the comparison by dividing the points into accuracy intervals are presented. The proportion of the points that fall within the set accuracy is summarized in Table A2.

Based on Table A1 and Table A2, it can be concluded that the data not subjected to post-processing have lower accuracy. In their case, about 60% of the points achieve an accuracy of 6 cm or less, and about 90% reach up to 9 cm. In contrast, the Pix4D Depth and Pix4D Fused data show much better precision—for both methods, about 50% of the points are within 1 cm, and as many as 95% of the points reach an accuracy of up to 3 cm.

The density of points in the cloud is also an important factor in assessing the usability of the data. The density was calculated on a circle with a radius of 1 cm; the results are as follows: cloud TLS—177 points; 3DScannerApp—3 points; Pix4D Captured—7 points; Pix4D Depth—201 points; and Pix4D Fused—104 points.

**Figure 11:**
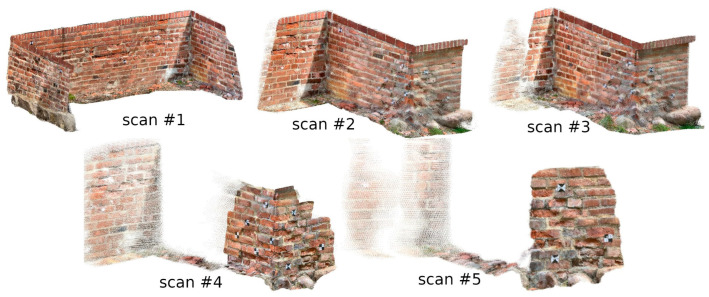
Measurement results from the SiteScape application.

Similar analyses were conducted for measured data of the city wall fragments with significant erosion. When measuring the D and M fragments (using the SiteScape application), the problem with the limited number of points in the cloud arose again. Therefore, five scans were made with this application (two scans for the other methods), changing the speed of the operator. In Figure 11, the point clouds acquired with this application are shown. Clouds 1–3 were acquired from a distance of about 0.8 m, while clouds 4 and 5 were acquired at about 0.3 m.

The first parameter studied was the density of individual point clouds. The density is expressed as the average number of points located in a sphere with a radius of 1 cm, and the results are shown in Table 1, which shows that the clouds representing fragment D have a lower density, which is directly related to the distance from which they were acquired. It is worth noting that both clouds acquired with 3DScannerApp have a density of about 4 points/cm^2^. This suggests that during post-processing, the app uses a generalization algorithm that calculates the position of points based on multiple observations. In addition, a very high density of point clouds from the SiteScape app is also notable. This is due to the lack of generalization—every point that has been measured is exported without further processing, resulting in significant data noise.

The data were then imported into a common coordinate system. The distances between the reference cloud and the test clouds were calculated on the sets prepared in this way. The results of this analysis are shown in Table A3.

Based on Table A3, it can be seen that the approach of measuring smaller city wall fragments yielded better results than measuring the whole object. For fragment D, a measurement accuracy up to 5 mm was achieved for 37.54, 19.01, 83.29, and 97.42% of points in 3DScannerApp, Pix4D Captured, Pix4D Depth, and Pix4D Fused, respectively. For the M fragment, this accuracy was achieved for 80.86, 78.00, 92.94, and 98.55% of the points, respectively. The same trend as for the whole wall can be seen here—the post-processed data show significantly higher accuracy.

The SiteScape application achieved an accuracy of less than 5 mm for between 14.56 and 50.18% of points, depending on the measurement. In addition to data size limitations, the app also stands out because of its different measurement method. In 3DScannerApp and Pix4D, points can be measured multiple times, and their coordinates are averaged. The exact algorithm that determines the correct measurement value is not known, but it has been noted that the longer a fragment is scanned, the more adjusted the measurements become. In the case of the SiteScape application, there is no averaging algorithm—all the measured points are exported. This results in a much denser point cloud but also leads to more noise.

For the smallest measured fragment M, the accuracy analysis results are the most favorable. An accuracy of 5 mm was achieved by more than 78% of the points in all variants of 3DScannerApp and Pix4D. For the Pix4D Fused cloud, more than half of the points achieved an accuracy of 2 mm.

### 4.2. Stage 2

**Figure 12:**
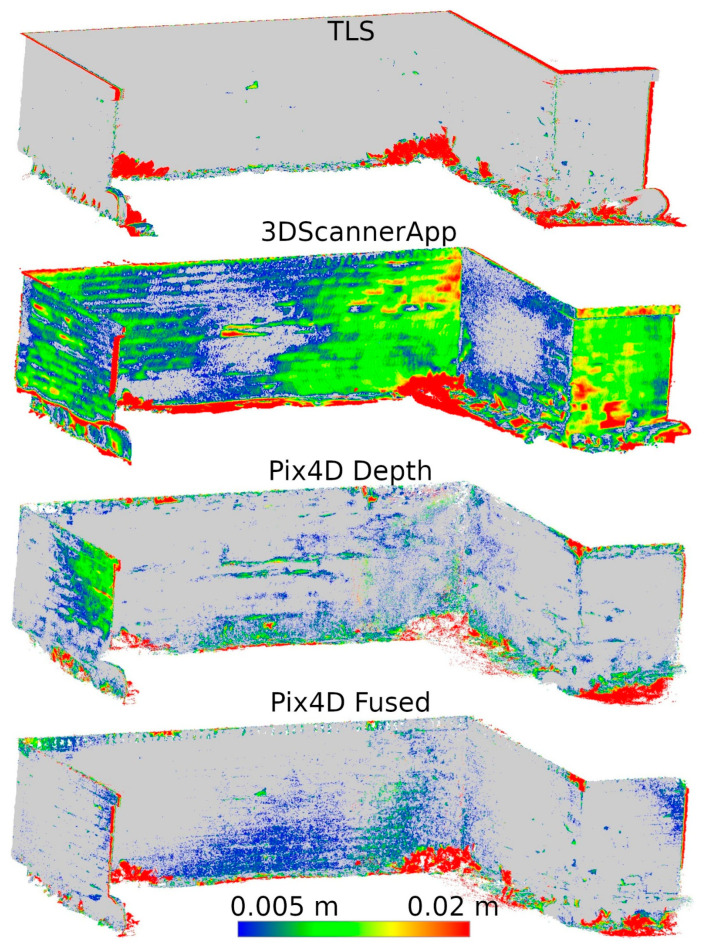
Differences between Stages 1 and 2 for city wall fragment D.

**Figure 13:**
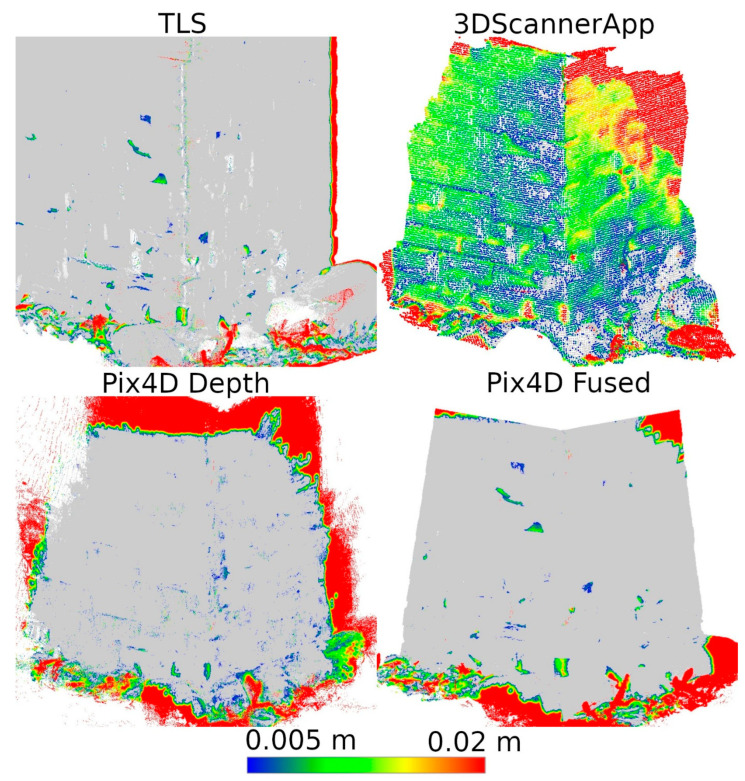
Differences between Stages 1 and 2 for city wall fragment M.

Based on the analyses conducted in Stage 1 of this study, a selection of data sources was made to be used in Stage 2. During the second measurement, data from SiteScape and Pix4DCatch Captured applications were eliminated, as both of these sources demonstrated the lowest accuracy. The remaining data sets were compared in terms of their ability to determine the degree of degradation of the site. Distances between the data of Stages 1 and 2 from the same sources were calculated. The results for fragments D and M are shown in Figure 12 and Figure 13, respectively.

For better visualization, the scale was set to minimum and maximum values of 0.005 and 0.02 m, respectively. Differences less than 0.005 m were marked in gray, while differences larger than 0.02 m were taken as maximum scale values and marked in the corresponding color. The large number of maximum differences at the cloud periphery is due to differences in cloud boundaries from Stages 1 and 2. In contrast, the maximum differences at the bottom of the wall were due to changing vegetation that developed between measurements. Both of these phenomena do not significantly affect the evaluation of the entire cloud, as they occur outside the main areas of interest.

Based on Figure 12, it can be concluded that Pix4D Fused, Pix4D Depth, and 3DScannerApp have the closest results to TLS, respectively. Due to the size of the city wall fragment, the deviations in the low-cost LiDAR measurement technology are clearly larger than those of TLS or photogrammetry-supported LiDAR. Similar results were also obtained during Stage 1, where for fragment D, the accuracy of 3DScannerApp was the lowest among the methods used in Stage 2.

Analyzing Figure 13, we see that the 3DScannerApp app gives more accurate results for a smaller fragment. The differences between the data from this app and TLS are larger, but where the TLS data show cavities, similar changes are also seen in the 3DScannerApp data. The larger differences at the periphery of the data are due to fewer observations in these areas during measurement.

**Figure 14:**
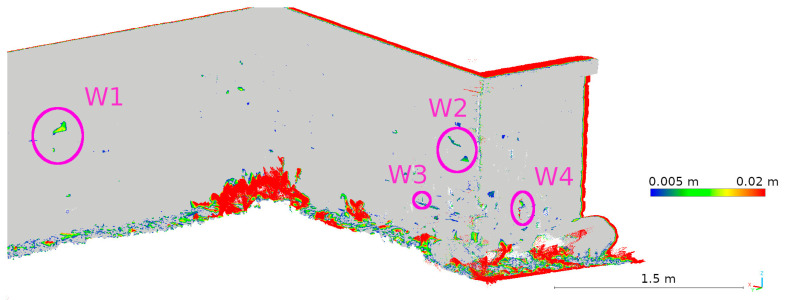
Location of selected defects where degradation has occurred.

For a more detailed analysis, four defects were selected to represent the cavities seen in the TLS data. The locations of these defects are shown in Figure 14. Defect W1 was used to analyze data from a larger fragment of the wall (D), while defects W2, W3, and W4 were analyzed on data from a smaller fragment of the wall (M).

Two data presentation methods were used to analyze the selected defects. The first was to rasterize the cloud slice on a plane to obtain a raster with a resolution of 1 mm per pixel. The pixel values were interpolated from the point cloud. The number of points from which the interpolation was performed is shown on each raster. 

**Figure 15:**
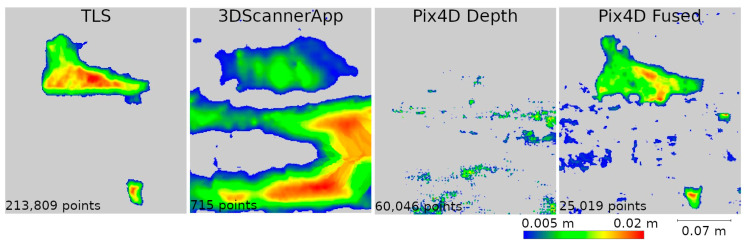
Defect W1 projected onto the plane.

**Figure 16:**
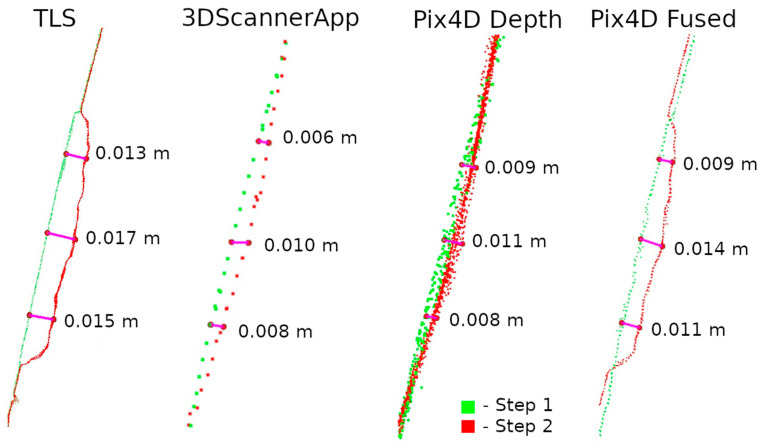
Cross-sections through defect W1.

**Figure 17:**
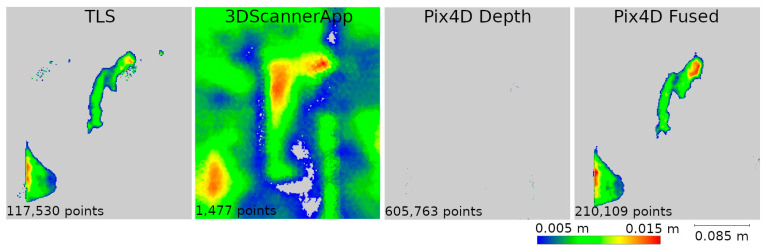
W2 defect projected onto the plane.

**Figure 18:**
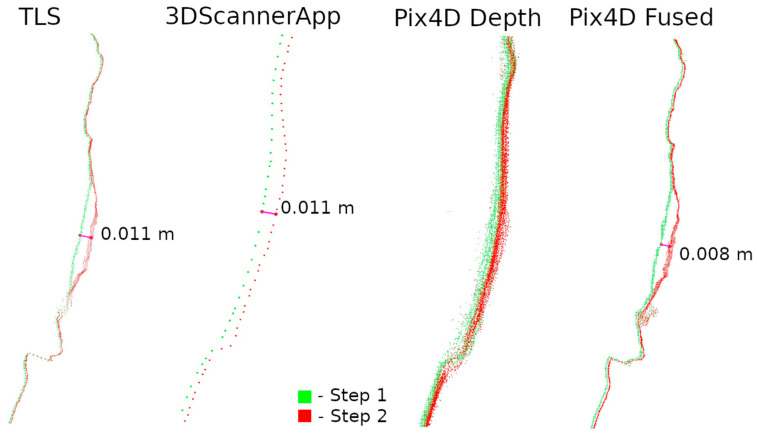
Cross-sections through defect W2.

**Figure 19:**
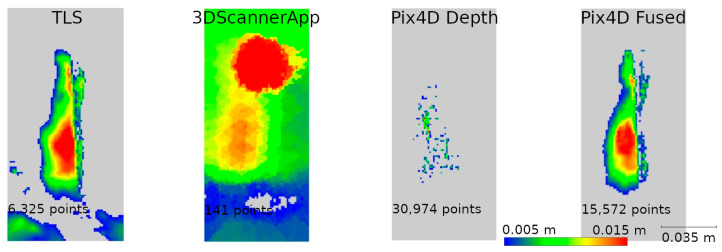
W3 defect projected onto the plane.

**Figure 20:**
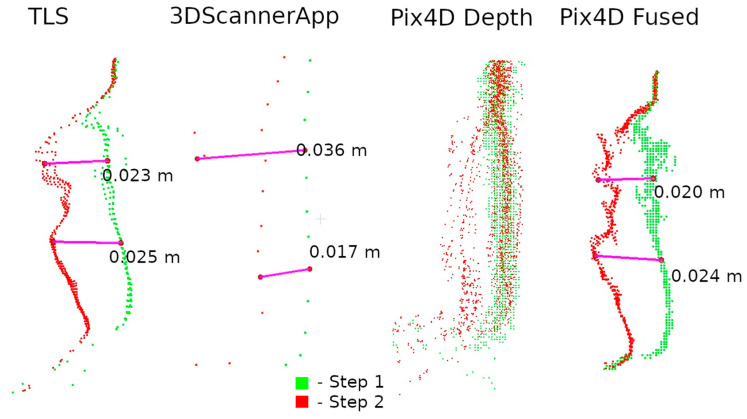
Cross-sections through defect W3.

**Figure 21:**
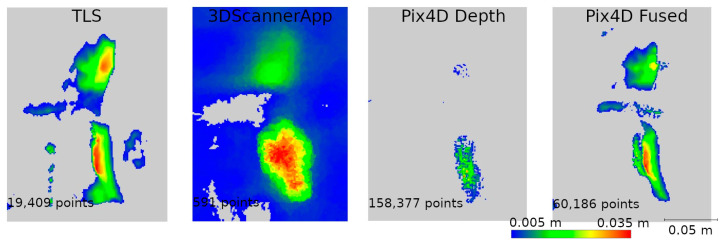
W4 defect projected onto the plane.

**Figure 22:**
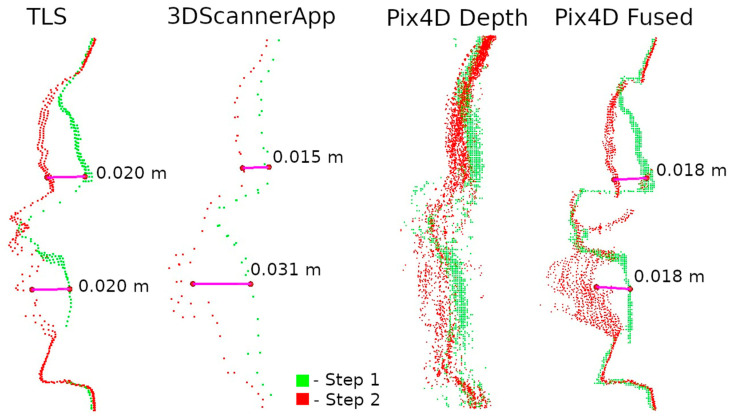
Cross-sections through defect W4.

The second method of presenting the results was to make a cross-section through the point cloud. For each defect, a cross-section of 0.005 m was made, positioned to contain as much information as possible. Horizontal cross-sections were made for defects W1 and W2, while vertical cross-sections were made for defects W3 and W4. The distances between points were marked on the cross-sections in an effort to have them in corresponding locations. Raster and cross-sections for each defect are shown in pairs to facilitate interpretation. For defect W1, the raster is shown in Figure 15 and the cross-section is shown in Figure 16. Similarly for the other defects W2 (Figure 17 and Figure 18), W3 (Figure 19 and Figure 20), W4 (Figure 21 and Figure 22).

Upon analyzing the various data sources, Figure 15 shows that 3DScannerApp, in addition to the attrition seen in the TLS data, also presents a significant attrition below that does not actually exist. This is an obvious error caused by the low accuracy of the data. Large measurement errors in this defect can also be seen in Figure 12. The defect detected according to the TLS data has a different shape, which is due to the difference in the number of points from which the raster was interpolated in 3DScannerApp compared to TLS.

The Pix4D Depth data showed virtually no attrition in this defect, while the Pix4D Fused data presented attrition that largely matched the TLS data. The reasons for the misrepresentation of the data in Pix4D Depth can be seen in Figure 16. The noise level is so high that information about potential loss is lost. The cross-section also shows the much lower density of data from 3DScannerApp. In Figure 16, one can see that the shape of the defect in the Pix4D Fused data is very similar to that from TLS, but there is an offset of 4 mm caused by fitting the clouds into a single coordinate system.

The remaining defects (W2, W3, and W4) are from the fragment M point cloud. In all previous analyses, it was characterized by better accuracy for low-volume data sources than the fragment D cloud, which is confirmed here as well. It can already be seen in Figure 13 that the defects defined as cavities in Figure 14 are also visible in the 3DScannerApp and Pix4D Fused data. In the case of the Pix4D Depth data, the degree of graying resulted in the loss of defect information in all three defects.

In the analyses shown in Figure 17, Figure 19, and Figure 21, the data from Pix4D Fused performed best. Nevertheless, the data directly from the low-cost scanner also showed cavities in the same locations where they were detected by TLS. Although the shape of these cavities may differ from the reference data, this is mainly due to the fact that the rasters were interpolated from a smaller number of points.

Analyzing the defects seen in Figure 18, Figure 20, and Figure 22, we note that, in each of the three cases, the defect is visible in the 3DScannerApp data. Its values vary from defect to defect: for defect W2, the manually measured distances differ by 0.007 m; for W3, they are consistent with the reference data; and for W4, the differences are 0.0013 and 0.008 m. These differences are directly related to the accuracy of determining the position of the points, which was investigated in Step 1 and is shown in Table A1.

One can also see in the cross-sections why the Pix4D Depth data do not show cavities in the rasters. This is due to the significant noise in the data. Distances between point clouds were calculated using the nearest neighbor algorithm. Due to the high noise, the nearest point is often a point of noise, making it too close to the examined point from Step 1.

An additional element of the analysis was to explore the possibility of using a handheld scanner to study the degradation of a historic object, using a fragment of the city walls as an example. The measurement process was analogous to the previous measurement methods (except for the measurement of the entire wall). Accordingly, only the D and M wall fragments were scanned (Figure 8). The measurement by this method was performed only during Stage 2 of measurement, so the analyses of the obtained results are presented separately. Accuracy analysis was carried out according to the same criteria as for the rest of the data in Stage 1.

The average density of point clouds in a sphere-shaped area with a radius of 1 cm was calculated. The Shining D cloud had an average density of 259 points, while that for the Shining M cloud was 2027 points. It is worth noting, however, that unlike the other low-cost methods, the handheld scanner allows the operator to control the density of the measurement, just as in the case of terrestrial scanners. The values given are for the clouds acquired for the purpose of this article, but it is possible to change the density during measurement depending on research needs.

The next step was to determine the accuracy of object mapping using this method. For this purpose, the distances between the TLS cloud from Step 1 and the Shining D and M clouds were calculated, and the results were classified and are presented in Table A4. As can be seen, the handheld scanner showed similar correlations to the other low-cost methods. The cloud describing fragment D is characterized by lower accuracy, where 76.60% of the points reach an accuracy of up to 20 mm. On the other hand, for the cloud describing fragment M, 76.59% of the points reach an accuracy of up to 3 mm.

**Figure 23:**
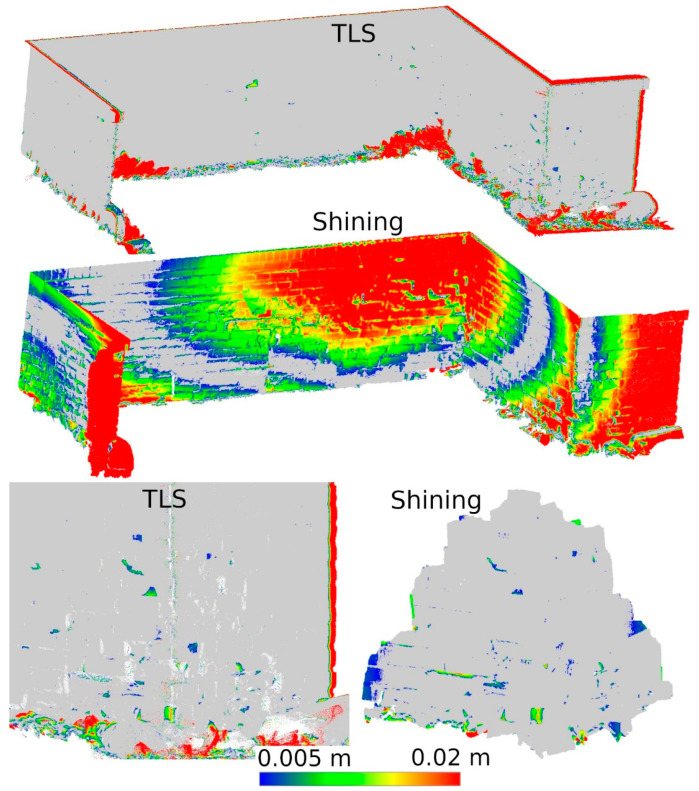
Differences between Stages 1 and 2 for measurements taken with a handheld scanner.

In order to detect potential cavities, a difference analysis was performed between the TLS cloud from Stage 1 and the Shining D and M clouds from Stage 2. The difference in approach from the other methods was due to the absence of the point cloud measured with the handheld scanner in Stage 1. The results of this analysis, showing the differences obtained, are presented in Figure 23. In the case of fragment D, clear anomalies can be seen, as well as significant cavities in places where they should not be. This is an obvious measurement error, which makes the data not useful in assessing the degradation of the object. In contrast, in the case of fragment M, the results obtained are almost identical to the TLS reference results. Since the point cloud for fragment D showed significant measurement errors during the initial analysis, it was not considered for further analysis.

**Figure 24:**
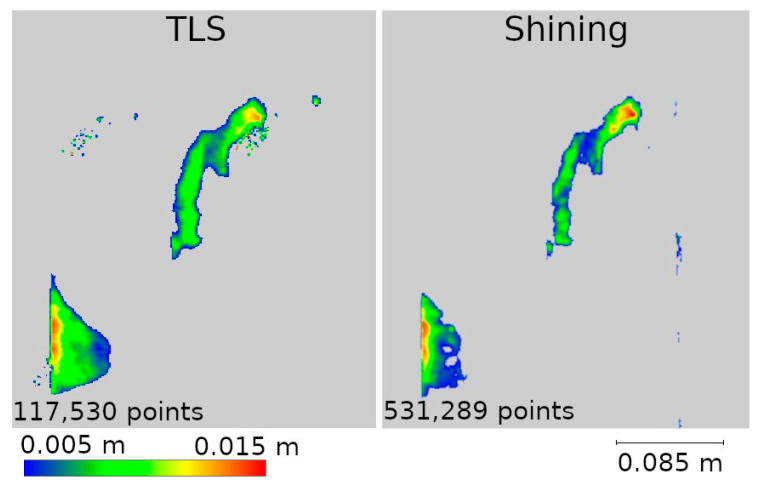
Defect W2 projected onto the plane—handheld scanner.

**Figure 25:**
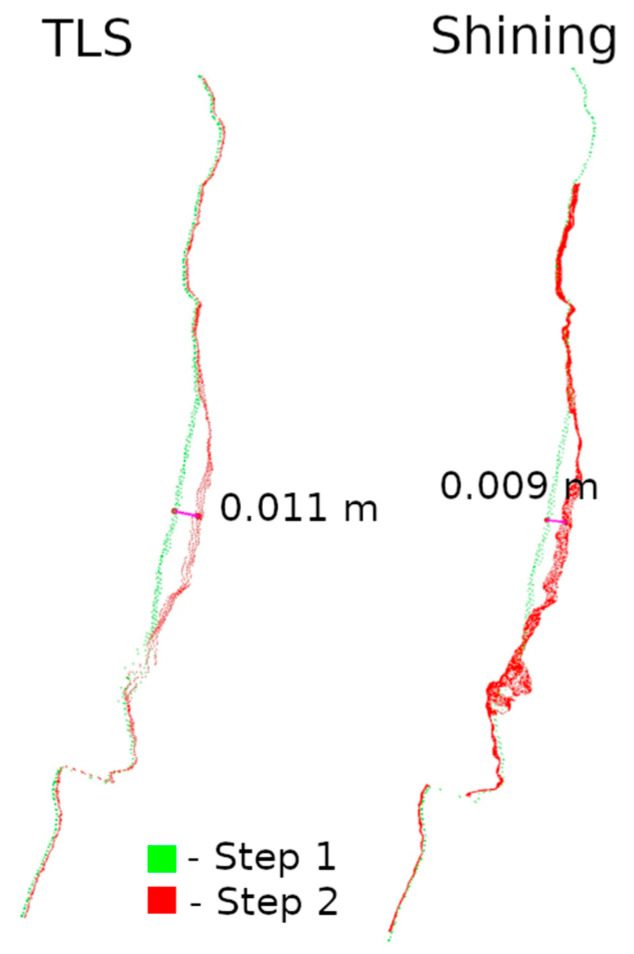
Cross-sections through defect W2—handheld scanner.

**Figure 26:**
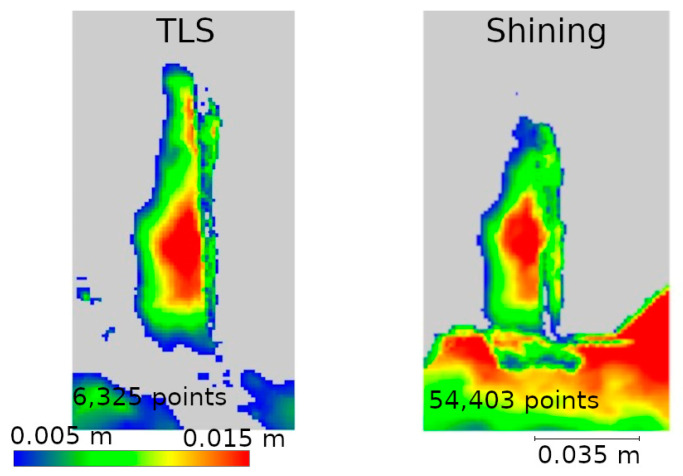
Defect W3 projected onto the plane—handheld scanner.

**Figure 27:**
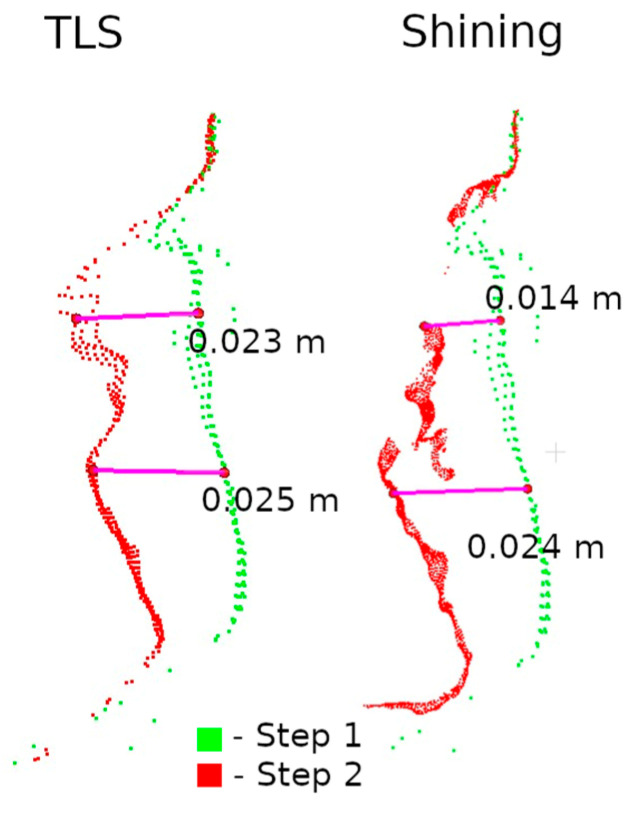
Cross-sections through defect W3—handheld scanner.

**Figure 28:**
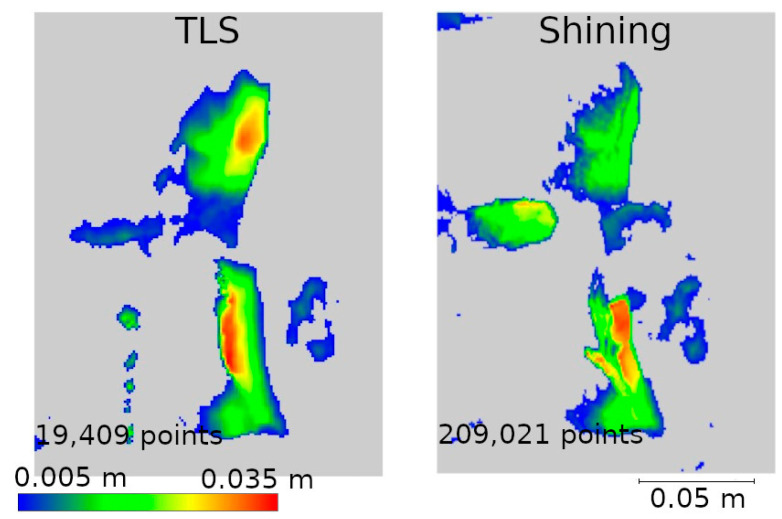
Defect W4 projected onto the plane—handheld scanner.

**Figure 29:**
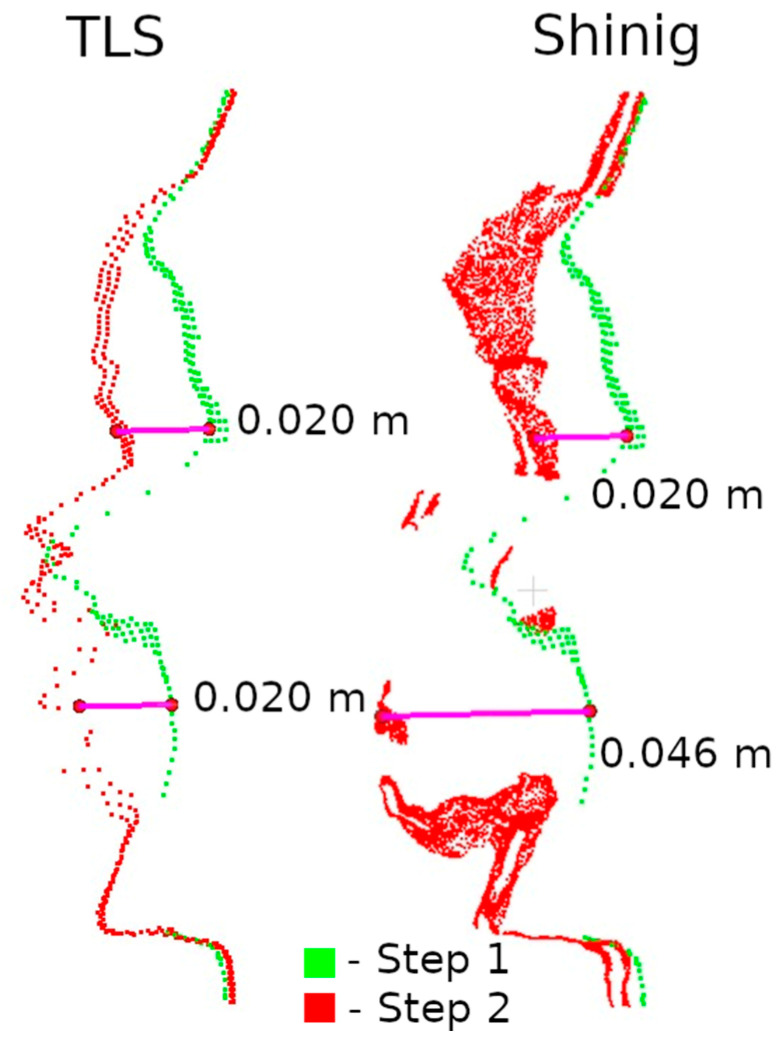
Cross-sections through defect W4—handheld scanner.

Raster and cross-sections were made for defects W2, W3, and W4 (Figure 14). The results are presented in pairs of raster and cross-section for each defect: W2 (Figure 24 and Figure 25), W3 (Figure 26 and Figure 27) W4 (Figure 28 and Figure 29). Analyzing the obtained rasters, we see that they are very close to the reference rasters. The slight differences seen in the upper part of Figure 26 are due to the characteristics of the measurement with the handheld scanner. The degree of insolation has a strong influence on the quality of measurement with this device. In a situation where one part of the city walls is sunlit well and another remains in shadow, measurement gaps can occur, which was the case here.

At its bottom, another case can also be observed. The differences are due to the greater flexibility of measurement with the handheld scanner. The W3 defect is located relatively low, as can be seen in Figure 14. Consequently, the terrestrial scanner, taking the measurement from a height of about 1.70 m, had this defect partially obscured by the brick above, making it impossible to map it correctly. The handheld scanner, due to its mobility, had access to this part of the wall and correctly detected the defect.

## 5. Discussion

Inventory surveys to determine the degree of deterioration of historic buildings are crucial from the perspective of keeping them in good condition. These structures often deteriorate due to underinvestment, which leads to their gradual degradation. The ability to take low-cost and accurate measurements can help historic preservationists monitor the condition of buildings, which in turn enables a faster response to progressive damage. The main purpose of this research program was to explore the possibility of using low-cost LiDAR technology for 3D modeling and degradation assessment of historic objects.

As this study showed, the accuracy of the obtained point clouds is affected by the following factors:

- Size of the measured object;
- The shape of the measured object;
- The distance from which the measurement is made;
- The application used.

The effect of the size of the object to be measured on the accuracy of the measurement was noted earlier by Teppati Losè et al. [13], among others, showing in their study that low-cost measuring devices will work well for measuring small- and medium-sized objects. The analyses conducted in the current study also prove this. The iPad scanner does not effectively measure the entire wall marked as 2 in Figure 3. The attempts we made did not yield the expected results, and depending on the application used, measurement errors ranged from a few to several centimeters (see Table A1). The largest deviations occurred at the points where measurements began and ended. The reason for these errors can be traced to the algorithm responsible for matching successive measurement scenes to a common coordinate system. In the case of stationary scanners, the center of the measurement system remains static throughout the measurement from a single station, and the measured points are positioned relative to a single common center of the system. With mobile scanners, the situation is different—the measuring device is in constant motion, which means that each successive series of measurements refers to a different location in space. The real-time registration algorithms, which are responsible for fitting these measurement series into a single coordinate system, are based on finding common parts in two adjacent images and matching them. This process is repeated as many times as the device sends out laser beams to measure the next scene. Errors resulting from the incorrect matching of neighboring imaging accumulate with each subsequent match. The longer the measurement (the larger the object), the more imaging the algorithm must combine, increasing the risk of significant measurement errors. Application manufacturers do not provide users with information on the algorithms used, and it is the quality of the algorithms, in addition to the accuracy of the sensor itself, that has a key impact on the quality of the final product.

**Figure 30:**
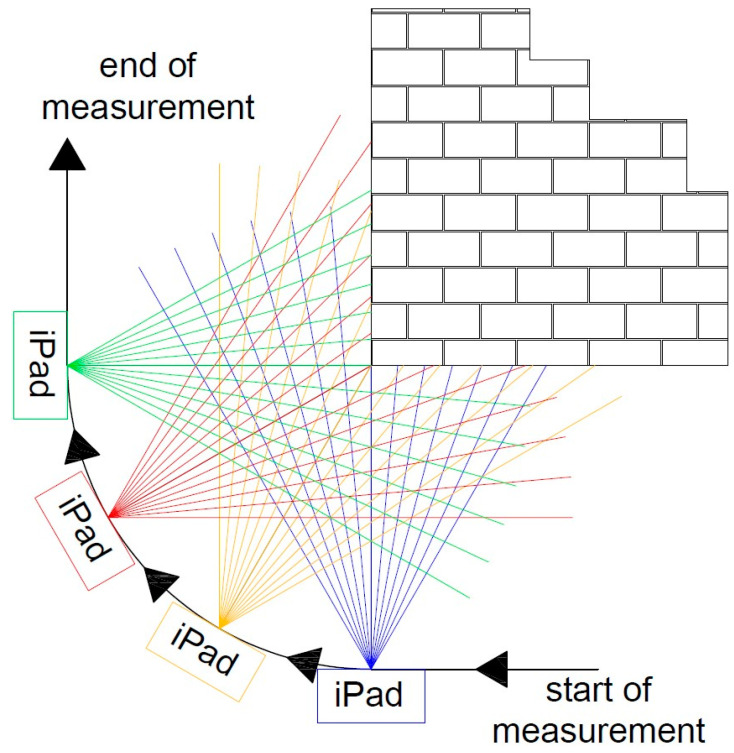
Example path of a single measurement with marked sample positions of the device.

**Figure 31:**
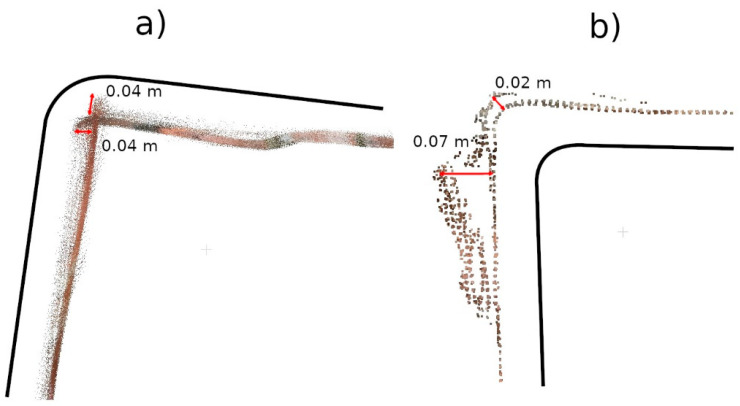
Examples of errors created at corners with the device’s trajectory marked: (**a**). SiteScape; (**b**). 3DScannerApp.

The under-occupation of the point clouds seen in Figure 10 draws attention to the second important factor, which is the shape of the object. Low-cost applications have difficulty accurately representing the angles between two planes. The more corners a measured feature has, the greater the likelihood of error accumulation. This phenomenon is particularly evident in data that have not been post-processed (Pix4D Capture, SiteScape) and in data where post-processing has been carried out directly on a mobile device (3DScannerApp). The main reason for this phenomenon is the angle of incidence of the laser beam on the plane, as noted by other researchers [15,16]. Figure 30 shows a diagram of the measurement of a corner of an object, with the four positions of the measuring device marked, and it can be seen that when measuring a corner, there comes a point when the device moves along the arc, starting to measure the other wall of the building, but at a high angle of incidence of the laser beam. When the device is in the middle of the arc, both walls are measured at an acute angle. In the last phase, the device measures at an acute angle the wall that was already measured earlier. Such measurement conditions lead to the generation of a large number of erroneous measurement points, which become noise and should be filtered out in post-processing. At the same time, erroneous measurements can interfere with the algorithm responsible for matching subsequent measurements to a common coordinate system. As a result, the algorithms may misrepresent the corner angle, and, in some cases, a double wall may even be imaged, as shown in Figure 31. In some situations, it is sufficient to clear the cloud of erroneous observations, as in Figure 31b. Nevertheless, in other cases, the corner angle is mapped incorrectly, rendering such data useless, as shown in Figure 31a.

In addition to the angle of incidence of the laser beam, the distance from which the data are acquired also has a significant impact on the accuracy of the measurements. The maximum range of the scanner installed on the iPad is 5 m, but the shorter the distance, the greater the accuracy that can be obtained. For a handheld scanner, the measurement distance is even shorter at 0.3 m. In the research presented here, the entire wall was measured from a distance of 1 m, the D fragment from 0.8 m, and the M fragment from 0.3 m. Analyses of the accuracy of the point clouds clearly indicate that the highest accuracy was achieved for the smallest fragment of the wall, which was measured from a close distance. The main reason for this is the amount of acquired measurement data—the shorter the distance from the object, the smaller the area onto which the grid of measurement points is projected, resulting in a higher point density and accuracy. This relationship was investigated by Spreafico et al. [23].

The last important factor for a LiDAR scanner mounted on a mobile device is the choice of application. This study presents the results of three selected apps, 3DScannerApp, SiteScape, and Pix4DCatch, each of which featured a different approach to measurements. The SiteScape application proved to be the least useful in the context of inventory measurements. Its main problem is the lack of point generalization. All measured observations are exported as a raw point cloud, with no pre-processing in the application. This leads to a lot of data noise, which negatively affects the accuracy of measurements. This phenomenon has also been noted by other researchers, such as Teppati Losè et al. [13] and Vacca [10]. In most of the measurements made, about 80% of the points reached an accuracy of 1.5 cm relative to the TLS. Another limitation is the maximum size of the acquired point cloud, which made it impossible for the application to measure the selected section of the wall according to the assumed parameters (distance from the object and measurement speed). Another application, 3DScannerApp, was the only free application used in the research, which worked well for the inventory of the city walls. When checking the accuracy in Stage 1, about 80% of the points were obtained with an accuracy of up to 15 mm for fragment D, with about 80% obtained with an accuracy of less than 5 mm for fragment M. Both these results and the analyses conducted in Stage 2, seen in Figure 12 and Figure 13, show that the app does its job, but only for small areas (fragment D had an area of about 13 m^2^, while that for fragment M was 1.5 m^2^). In addition to the area measured, it is also important to note the size of the cavities. The cavities surveyed on fragment M were no less than 2 cm wide and between 1 and 3 cm deep. Cavities of this size were correctly detected, as can be seen in Figure 17, Figure 19, and Figure 21. Despite the correct detection of the defect, accurately determining its size could be problematic due to the limited accuracy of point positioning. The cross-sections seen in Figure 18, Figure 20, and Figure 22 show that for the W2 defect, the measured distances differ by 0.007 m; for W3, they are consistent with the reference data; and for W4, they are 0.0013 and 0.008 m, respectively. These differences are due to both the accuracy of the measurements and the density of the point cloud. In some cases, it was impossible to select two corresponding points on the reference and test clouds due to the lower point cloud density in the test data. The last application used was Pix4DCatch, which was used to obtain three data sets: Captured, Depth, and Fused. The first is a direct product of the mobile application and requires no further elaboration. The results of this data set in Stage 1 were similar to those of the 3DScannerApp, but an account with an active Pix4D product is required to export these data, making the app no longer low-cost for most users. As a result, the use of these data in Step 2 was abandoned. The Depth set is the result of processing LiDAR scanner data in the Pix4Dmatic application, while the Fused data are a combination of LiDAR data and short-range photogrammetry. Analyses showed that the Depth data, despite being developed in the desktop application, are characterized by significant noise, making it unsuitable for defect detection, as can be seen in Figure 17, Figure 19, and Figure 21. The Fused data set proved to be the most effective of all those analyzed. It achieved an accuracy of 90% of points within a 2 mm error. Stage 2 analyses showed that the geometric representation of the object is comparable to the results obtained with the TLS scanner. The process of acquiring Fused data is as simple as with the other applications, but the need to process them in a desktop application significantly increases the cost. Nevertheless, the cost is still significantly lower than that of purchasing a TLS scanner.

The accuracy of the data acquired with the FreeScan handheld scanner and its suitability for historic site inventory are comparable and sometimes even superior to the terrestrial scanner (provided a small area is measured). All analyses of data from the handheld scanner for fragment M show that its accuracy matches that of the terrestrial scanner. In addition to the high precision of point positioning, the device has the added advantage of mobility. Thanks to the measurement method, the user can map the entire fragment as a single measurement approach, which, in the case of TLS, would require the use of several stations from different angles. In some cases, such a solution would be very difficult, such as when measuring cavities close to the ground or at heights beyond the device’s range. The biggest disadvantage of a handheld LiDAR scanner is its susceptibility to varying lighting conditions. With large differences in lighting, the device may fail to register shaded fragments, affecting the quality of the measurement.

## 6. Conclusions

1. Low-cost LiDAR scanners can be successfully used to detect cavities in historic architectural structures, enabling their continuous monitoring. The low cost and ease of use of this technology enable cyclic measurements to be conducted even in small time intervals. However, the effectiveness of this technology is closely related to the size of the measured area. In this work, it was shown that this technology gives the best results on an area of about 1.5 m^2^, allowing the detection of cavities at least 2 cm wide and 1 cm or more deep.
2. The most precise results were obtained with more advanced measurement methods. Both the data acquired with the Pix4DCatch application, subsequently developed in the Pix4Dmatic desktop application, and the data from Shining 3D’s FreeScan handheld scanner showed an accuracy comparable to TLS technology. This demonstrates the significant development of LiDAR technology, which makes it possible to obtain accurate point clouds at a lower cost, but free apps and mobile devices still cannot match the precision of TLS scanners.
3. Due to its mobility, the handheld scanner and the Pix4DCatch app prove to be a better solution in situations where the inventory covers a small part of the monument. In the context of such applications, the mobility and relatively high precision of these tools make them more practical than desktop TLS systems.

## Figures and Tables

**Table 1:** Density of the point cloud expressed in number of points per circle of radius 1 cm.

Data	Number of Points
TLS	191
3DScannerApp D	3.5
3DScannerApp M	4
Pix4D Captured D	4
Pix4D Captured M	62
Pix4D Depth D	86
Pix4D Depth M	1564
Pix4D Fused D	45
Pix4D Fused M	555
Sitescape 1	258
Sitescape 2	298
Sitescape 3	215
Sitescape 4	1508.8
Sitescape 5	1224

## Data Availability

The original contributions presented in the study are included in the article, further inquiries can be directed to the corresponding author.

## Appendix A

**Table A1:** The number of cloud points located at a given distance from the reference cloud. Data for the entire section of the wall. Red—0–5%; yellow—5–10%; green—10–15%; blue—15–20%; white—the rest.

	<1 cm	1–2 cm	2–3 cm	3–4 cm	4–5 cm	5–6 cm	6–7 cm	7–8 cm	8–9 cm	9–10 cm	10–15 cm	15–20 cm	20–25 cm	25–30 cm	30–35 cm	35–40 cm	40–45 cm	>45 cm
3dScannerApp	27.72%	18.79%	9.88%	9.06%	7.73%	7.79%	3.71%	2.02%	1.77%	1.35%	2.54%	1.75%	1.36%	1.20%	1.03%	1.07%	0.97%	0.25%
Pix4D Captured	26.50%	16.71%	11.39%	8.93%	8.03%	6.14%	5.22%	4.58%	2.89%	1.78%	5.89%	1.00%	0.46%	0.21%	0.12%	0.06%	0.05%	0.03%
Pix4D Depth	50.03%	36.51%	8.93%	0.68%	0.32%	0.27%	0.20%	0.21%	0.20%	0.19%	0.68%	0.48%	0.39%	0.33%	0.28%	0.16%	0.10%	0.06%
Pix4D Fused	52.45%	31.57%	10.52%	1.30%	0.51%	0.39%	0.32%	0.25%	0.21%	0.17%	0.62%	0.44%	0.35%	0.27%	0.29%	0.15%	0.10%	0.07%

**Table A2:** The number of points falling within the specified accuracy range. Data for the entire section of the wall. Green—25–50%; yellow—50–75%; red—75–95%; white—the rest.

	<1 cm	<2 cm	<3 cm	<4 cm	<5 cm	<6 cm	<7 cm	<8 cm	<9 cm	<10 cm	<15 cm	<20 cm	<25 cm	<30 cm	<35 cm	<40 cm	<45 cm	>45 cm
3dScannerApp	27.72%	46.52%	56.40%	65.45%	73.19%	80.98%	84.69%	86.71%	88.49%	89.83%	92.38%	94.13%	95.48%	96.68%	97.71%	98.78%	99.75%	100.00%
Pix4D Captured	26.50%	43.20%	54.59%	63.52%	71.55%	77.69%	82.92%	87.50%	90.38%	92.16%	98.05%	99.05%	99.51%	99.73%	99.85%	99.91%	99.97%	100.00%
Pix4D Depth	50.03%	86.53%	95.46%	96.14%	96.46%	96.73%	96.93%	97.14%	97.34%	97.52%	98.20%	98.68%	99.08%	99.40%	99.68%	99.84%	99.94%	100.00%
Pix4D Fused	52.45%	84.02%	94.54%	95.84%	96.35%	96.74%	97.06%	97.31%	97.52%	97.70%	98.32%	98.76%	99.11%	99.38%	99.67%	99.82%	99.93%	100.00%

**Table A3:** The number of points falling within the specified accuracy range. Data for fragments D and M. Blue—0–25%; green—25–50%; yellow—50–75%; red—75–95%; white—the rest.

	<1 mm	<2 mm	<3 mm	<4 mm	<5 mm	<6 mm	<7 mm	<8 mm	<9 mm	<10 mm	<15 mm	<20 mm	<25 mm	<30 mm	<35 mm	<40 mm	<45 mm	>45 mm
3DScannerApp D	3.48%	12.04%	21.48%	29.32%	37.54%	43.81%	50.46%	55.86%	61.37%	65.38%	81.69%	93.50%	99.29%	99.61%	99.76%	99.87%	99.94%	100.00%
3DScannerApp M	10.41%	37.82%	56.76%	71.16%	80.86%	86.73%	90.76%	93.40%	94.93%	96.04%	98.30%	99.15%	99.52%	99.72%	99.88%	99.98%	100.00%	100.00%
Pix4D Captured D	1.91%	5.93%	10.79%	14.31%	19.01%	22.64%	27.80%	31.80%	37.30%	41.57%	66.95%	84.44%	93.39%	96.53%	97.62%	97.94%	98.13%	100.00%
Pix4D Captured M	9.12%	34.76%	52.82%	67.91%	78.00%	85.12%	90.31%	93.48%	95.61%	97.09%	99.36%	99.79%	99.93%	99.98%	100.00%	100.00%	100.00%	100.00%
Pix4D Depth D	14.78%	40.32%	60.95%	73.61%	83.29%	88.81%	92.91%	95.20%	96.87%	97.81%	99.55%	99.87%	99.94%	99.98%	99.99%	100.00%	100.00%	100.00%
Pix4D Depth M	16.34%	52.32%	74.49%	86.19%	92.94%	96.31%	97.85%	98.74%	99.21%	99.45%	99.87%	99.96%	99.99%	100.00%	100.00%	100.00%	100.00%	100.00%
Pix4D Fused D	35.86%	76.47%	90.92%	95.59%	97.42%	98.29%	98.75%	99.04%	99.22%	99.35%	99.67%	99.83%	99.93%	99.97%	99.99%	99.99%	100.00%	100.00%
Pix4D Fused M	40.72%	90.79%	96.73%	97.99%	98.55%	98.89%	99.12%	99.28%	99.40%	99.49%	99.78%	99.93%	99.98%	100.00%	100.00%	100.00%	100.00%	100.00%
Sitescape 1	3.13%	9.22%	14.21%	18.65%	22.63%	26.17%	29.31%	32.09%	35.23%	37.60%	51.49%	67.43%	85.61%	97.06%	99.48%	99.78%	99.87%	100.00%
Sitescape 2	0.28%	3.73%	7.38%	11.12%	15.11%	19.44%	24.12%	29.17%	34.55%	40.17%	65.58%	84.09%	91.48%	95.53%	97.11%	98.01%	98.45%	100.00%
Sitescape 3	1.35%	4.71%	7.94%	11.20%	14.56%	19.82%	23.49%	27.28%	31.19%	35.23%	57.86%	75.88%	88.21%	95.38%	97.94%	98.81%	99.20%	100.00%
Sitescape 4	3.49%	9.76%	14.99%	19.89%	24.76%	29.85%	34.92%	39.53%	43.80%	48.04%	73.59%	90.02%	97.53%	98.71%	98.94%	99.07%	99.22%	100.00%
Sitescape 5	3.09%	12.83%	22.95%	34.12%	50.18%	62.06%	72.90%	81.75%	89.42%	92.88%	98.66%	99.52%	99.78%	99.89%	99.94%	99.96%	99.98%	100.00%

**Table A4:** The number of points falling within the specified accuracy range for the Shining data. Blue—0–25%; green—25–50%; yellow—50–75%; red—75–95%; white—other.

	<1 mm	<2 mm	<3 mm	<4 mm	<5 mm	<6 mm	<7 mm	<8 mm	<9 mm	<10 mm	<15 mm	<20 mm	<25 mm	<30 mm	<35 mm	<40 mm	<45 mm	>45 mm
Shining D	2.62%	6.37%	9.99%	17.29%	20.95%	24.58%	31.66%	35.02%	38.28%	44.39%	59.98%	76.60%	87.45%	94.00%	96.41%	97.55%	97.80%	100.00%
Shining M	24.64%	59.53%	76.59%	85.07%	89.06%	92.03%	94.04%	95.36%	96.53%	97.30%	98.69%	99.17%	99.57%	99.81%	99.90%	99.93%	99.95%	100.00%
